# Supplementary material for: Collaborative intelligence in AI: Evaluating the performance of a council of AIs on the USMLE
Source: PLOS Digit Health. 2025 Oct 9;4(10):e0000787. doi: 10.1371/journal.pdig.0000787 (PMC12510544; doi:10.1371/journal.pdig.0000787)
Supplement: S2 File — (ZIP) [file pdig.0000787.s002.zip › S2 Data Raw data files containing unprocessed question inputs and LLM outputs including transcripts of deliberations/STEP3_Question-77-1_Type-MC-NJ.docx]

**Facilitator** **[Round 1]:** A 34-year-old woman comes to the office because of a 3- to 4-week history of swelling of her legs and a 9-kg (20-lb) weight gain. Medical history is significant for sickle cell trait and mild anemia. She has been taking 800 mg of ibuprofen three times daily for Achilles tendinitis diagnosed 1 month ago. She has smoked five cigarettes daily for the past 15 years, and she drinks one to five beers on weekends. She experimented with cocaine briefly 16 years ago, but she has never used intravenous drugs. She has been in a monogamous sexual relationship for the past 12 years. Today, vital signs are temperature 37.2°C (99.0°F), pulse 88/min, respirations 16/min, and blood pressure 145/95 mm Hg. Physical examination discloses periorbital edema but no jugular venous distention. Lungs are clear to auscultation. Cardiac examination discloses an S1 and S2 without murmurs or gallops. Abdominal examination discloses bulging flanks and shifting dullness to percussion. Examination of the lower extremities shows pitting edema from the mid thigh to the ankles bilaterally. Results of which of the following studies are most likely to be abnormal in this patient? (A) Echocardiography (B) HIV antibody study (C) Serum B-type natriuretic peptide concentration (D) Toxicology screening of the urine (E) Urine protein concentration

**AI_C:** RESTATING THE QUESTION: A middle-aged woman came to the clinic with complaints of leg swelling and significant weight gain over the last 3-4 weeks. Her history includes sickle cell trait, mild anemia, and regular use of ibuprofen. Her lifestyle consists of smoking and weekend alcohol consumption. She had a brief history of cocaine usage 16 years ago and has an exclusive one partner relationship for the past 12 years. Her physical examination reveals signs indicative of fluid accumulation, such as periorbital edema and pitting edema but no signal of cardiac dysfunction. The question poses, given her condition and background, which test outcome between echocardiography, HIV study, Natriuretic peptide concentration, urine toxicology screening, or urine protein concentration, could be abnormal? PLANNING: The woman's presentation points towards systemic fluid overload. Factors to consider include her use of NSAIDs (like Ibuprofen that can cause fluid retention), sickle cell trait (though unlikely the cause of present symptoms), and her previous history of substance usage. While toxicity can't be ruled out completely given her history, her symptoms suggest a presence of renal pathology rather than cardiac or infectious diseases. CLINICAL REASONING: While this patient has a history of smoking and alcohol use, her symptoms of bilateral pitting lower limb edema, periorbital edema, along with bulging flanks and weight gain suggest systemic fluid overload. This makes renal-origin edema more likely, with proteinuria causing blood proteins to spill into the urine and decrease the oncotic pressure within the blood vessels, leading to the symptoms observed. The usage of NSAIDs exacerbates this as they can cause renal vasoconstriction and decreased glomerular filtration rate, leading to water and sodium retention. CONCLUDING: Given the above clinical reasoning, the study most likely to be abnormal in this woman is the urine protein concentration. The use of NSAIDs, the presence of sickle cell trait, and the signs of systemic fluid overload all suggest this probability. RESPONSE: The study most likely to deliver abnormal results given the current clinical scenario is the urine protein concentration (Choice E). The symptoms displayed by the woman are more strongly associated with proteinuria, a sign of renal pathology.

**AI_A:** (1) RESTATING THE QUESTION: The description portrays a 34-year-old female patient who has been experiencing a few weeks of lower limb swelling and rapid weight gain. Her past medical history is marked by sickle cell trait and mild anemia, uses ibuprofen for her Achilles tendinitis, smokes, drinks, and had a brief history with cocaine though no subsequent intravenous drug usage. Examination reveals signs of fluid accumulation in the body, but no abnormalities were found in cardiovascular and pulmonary assessments. With this clinical profile, we need to ascertain which test is more likely to yield abnormal results in this patient's situation. (2) PLANNING: The swelling (known as edema), weight gain, and dullness in percussion possibly indicate fluid build-up in her body, a condition called anasarca. The possible etiologies include cardiac, renal, or liver diseases. However, given her history of NSAID use, it's likely her kidney function may be impaired, leading to proteinuria and resulting in the observed edema. While the usage of illicit drugs raises questions about the possibility of HIV, her monogamous relationship and the period since her last drug use make this less likely. Given this, the clinical priority should be to check if the patient's symptoms are most probably due to a drug-induced kidney issue. (3) CLINICAL REASONING: Use of NSAIDs, like ibuprofen, is known to potentially affect the kidneys as one of their side effects. They can cause minimal change disease, a condition that presents with heavy proteinuria leading to nephrotic syndrome (edema, weight gain). Even though echocardiography could be important for cardiac evaluation, absence of murmurs, gallops, or jugular venous distention makes a cardiac cause for her edema less likely. Serum B-type natriuretic peptide concentration (which increases in heart failure) is less suggestive too due to lack of cardiac symptoms. Toxicology screening may not provide a rewarding output as she is distanced from heavy drug use. (4) CONCLUDING: Therefore, the woman’s symptoms of edema, weight gain, and high blood pressure seem to suggest a kidney issue possibly secondary to her ibuprofen use (given the circumstantial evidence). As such, the urine protein concentration test (urinalysis), which can indicate kidney damage if abnormal, should be the focus of our investigations. (5) RESPONSE: Hence, in line with the mentioned clinical thought process, the test which is most likely to be abnormal for this patient would be the 'Urine protein concentration' (Choice E). This will help in detecting proteinuria, which can be indicative of some form of kidney disease or damage.

**AI_B:** RESTATING THE QUESTION: We are given a scenario where a 34-year-old woman presents to a clinic with weight gain and leg swelling for about a month. Some of her main health history includes sickle cell trait, mild anemia, Achilles tendinitis, and ibuprofen use, along with smoking and occasional beer consumption. Her vital signs indicate slightly high blood pressure, and physical exam finding are suggestive of fluid accumulation in body cavities and tissues. We are then asked to determine which test might reveal abnormal results in this case. PLANNING: The main symptoms observed in the woman are weight gain and leg swelling, along with findings of periorbital edema and bulging flanks and shifting dullness to percussion on physical exam, these all point to fluid retention. In the absence of cardiac or pulmonary symptoms, these findings could suggest renal dysfunction. Importantly, her use of ibuprofen could have led to NSAID-induced interstitial nephritis, an inflammation of the kidneys which can result in proteinuria, a condition where the urine contains an abnormal amount of protein. The possibility of edematous pathologies like congestive heart failure or liver disease can be ruled out looking at her health history and cardiac and pulmonary clinical findings which were unremarkable. Other options like Echocardiography (used in cardiac diseases), HIV study (no significant risk factors noted), Serum B-type natriuretic peptide concentration (used in heart failure), Toxicology screening (no current substance abuse reported) might be less likely to demonstrate abnormal results in this case. CLINICAL REASONING: Periorbital edema, leg swelling, and shifting dullness to percussion all are clear indications of fluid overload, especially in the absence of jugular venous distention that would point toward a cardiac cause. Furthermore, the patient reported weight gain over the past month, which could be due to fluid retention secondary to a kidney condition. The recent regular use of ibuprofen, a non-steroidal anti-inflammatory (NSAID), could have induced interstitial nephritis, deteriorating kidney function and leading to proteinuria. CONCLUDING: Given the patient's recent persistent use of ibuprofen, along with the symptomatic findings of edema and a lack of evidence to suggest cardiac disease, this leads me to suspect NSAID-induced interstitial nephritis. This condition can be best confirmed through measuring abnormal serum protein levels in the urine. RESPONSE: Thus, urine protein concentration (E) is the test most likely to yield an abnormal result in this patient. The systemic edema, kidney-injurious drug use and her blood pressure suggest a renal pathology and the best initial test to confirm this would be checking for increased protein levels in the urine.

**AI_D:** RESTATING THE QUESTION: The query pertains to a 34-year-old female experiencing peripheral edema (leg swelling) and a significant weight gain over the previous 3-4 weeks. She has sickle cell trait and mild anemia in her medical history, and has been taking ibuprofen regularly to manage Achilles tendinitis. She is a moderate smoker and drinker, with a previous history of cocaine use. Her current vitals and physical examination reveal high blood pressure, periorbital edema (swelling around eyes), bulging flanks (indicating fluid accumulation), shifting dullness (suggestive of ascites which is abdominal fluid buildup) and pitting edema (presence of indentations in the lower extremity skin, indicative of fluid retention). The question is about which of the listed laboratory examinations (namely, Echocardiography, HIV antibody study, Serum B-type natriuretic peptide concentration, toxicology screening of the urine, or urine protein concentration) would likely yield unusual outcomes in her case. PLANNING: To determine which test results might be abnormal, I need to assess the potential causes of her symptoms in the context of her medical and lifestyle history. Key elements from the patient history include her history of ibuprofen use, smoking and drinking habits, hypertension, and her clinical signs of fluid overload. Firstly, the high blood pressure and her signs of edema could signify kidney dysfunction, potentially from long-term ibuprofen use which can lead to NSAID-induced nephropathy, causing abnormal protein leakage in the urine. Therefore, the urine protein concentration test could be abnormal. Alternatively, given her past cocaine use and current hypertension, an echocardiogram might show damage to the heart, even though other cardiac symptoms (like murmurs or gallops) were absent. Elevated B-type natriuretic peptide levels can indicate heart failure, but her history doesn't clearly suggest this. HIV antibodies and a toxicology screen could be abnormal due to her past drug use and sexual behavior but these factors don't relate directly to her symptoms. CLINICAL REASONING: Taking the priorities suggested by the plan into consideration, closer examination of her kidney function seems logical since the signs of fluid overload (including periorbital edema and leg swelling) and high blood pressure could be related to kidney damage, which can manifest from frequent and long-term use of NSAIDs like ibuprofen. An increase in urine protein would then indicate renal involvement, particularly, glomerular damage causing proteinuria. CONCLUDING: In connecting the steps of my thought process, given the presenting symptoms, medical history, and physical findings, an abnormal result is most likely to be obtained in the urine protein concentration test. This test will uncover potential kidney damage as a cause of her symptoms, which might have resulted from her extensive ibuprofen intake for Achilles tendinitis. RESPONSE: The most likely abnormal test result for this patient would likely be (E) Urine protein concentration, due to a potential kidney damage that might have been caused by long-term use of ibuprofen.

**AI_E:** RESTATING THE QUESTION: We are asked to identify the most likely abnormal investigation result in a 34-year-old woman who presents with a history of leg swelling and notable weight gain. The patient has a significant medical history of sickle cell trait and mild anemia. She regularly takes 800mg of ibuprofen for Achilles' tendinitis. She smokes, drinks, and she has a history of brief cocaine use 16 years ago, but hasn't engaged in intravenous drug use. She is in a stable sexual relationship and has been for the past 12 years. Upon physical examination, some notable findings include periorbital edema, bulging flanks, and pitting edema but there is no evidence of heart abnormalities or lung disease. PLANNING: The patient's presentation brings up a few key assumptions. First, the mention of the sickle cell trait points to the possibility of kidney disease as people with sickle cell trait are more likely to have problems related to kidney disease like hematuria and urinary concentration defects. Additionally, the increase in blood pressure and proximal edema could suggest kidney disease, probably nephrotic syndrome. This syndrome is characterized by free-floating proteins in the urine and edema because of protein loss through the urine. The edema is characterized as pitting, something often seen with nephrotic syndrome. Long term NSAID use, as in her ibuprofen use for the Achilles' tendinitis, can also lead to kidney disease. CLINICAL REASONING: The patient's history of mild anemia and sickle cell trait, coupled with her long-term NSAID use, present potential reasons for nephrotic syndrome to develop. This syndrome is commonly associated with edema and hypertension; both of which the patient has shown symptoms of. We have also observed edema and an increase in weight which correlate with fluid retention, another symptom of nephrotic syndrome. The mild anemia can also elevate the likelihood of glomerular damage leading to nephrotic syndrome. CONCLUDING: Given the moderation in her alcohol use, the smoking, the brief cocaine use years ago, the otherwise seemingly healthy lifestyle, and the present physical evidence, combined with the knowledge of her long-term ibuprofen use and sickle cell trait, nephrotic syndrome is evidently the most fitting explanation. RESPONSE: In order to validate the hypothesis that the patient is suffering from nephrotic syndrome, the aberrant results one would expect to find should be linked to kidney damage or dysfunction. An examination of the (E) Urine protein concentration would most likely yield abnormal results in this patient, as it would indicate a high level of protein in the urine commonly found in nephrotic syndrome. Other tests (A, B, C, D) are not as directly linked to the signs and symptoms of this patient, although an echocardiography may show minor anomalies due to the long-term pressure placed on the heart from her high blood pressure, these effects would be secondary and not primary as with kidney damage, function, and subsequent tests.

**Facilitator** The consensus among the AI responses is that the most likely abnormal test result for this 34-year-old woman, who presents with leg swelling and significant weight gain, would be the urine protein concentration (Choice E). This conclusion is based on her clinical profile, which includes a history of sickle cell trait, mild anemia, and regular use of ibuprofen. The patient's symptoms of edema, weight gain, and high blood pressure, along with her history of NSAID use, suggest a kidney issue possibly secondary to her ibuprofen use. The use of NSAIDs, like ibuprofen, is known to potentially affect the kidneys as one of their side effects. They can cause minimal change disease, a condition that presents with heavy proteinuria leading to nephrotic syndrome (edema, weight gain). Therefore, the urine protein concentration test, which can indicate kidney damage if abnormal, should be the focus of our investigations. Other tests such as echocardiography, HIV antibody study, serum B-type natriuretic peptide concentration, and toxicology screening of the urine are less likely to yield abnormal results given the patient's current symptoms and history.
